# Interval Neutrosophic Sets and Their Application in Multicriteria Decision Making Problems

**DOI:** 10.1155/2014/645953

**Published:** 2014-02-17

**Authors:** Hong-yu Zhang, Jian-qiang Wang, Xiao-hong Chen

**Affiliations:** School of Business, Central South University, Changsha 410083, China

## Abstract

As a generalization of fuzzy sets and intuitionistic fuzzy sets, neutrosophic sets have been developed to represent uncertain, imprecise, incomplete, and inconsistent information existing in the real world. And interval neutrosophic sets (INSs) have been proposed exactly to address issues with a set of numbers in the real unit interval, not just a specific number. However, there are fewer reliable operations for INSs, as well as the INS aggregation operators and decision making method. For this purpose, the operations for INSs are defined and a comparison approach is put forward based on the related research of interval valued intuitionistic fuzzy sets (IVIFSs) in this paper. On the basis of the operations and comparison approach, two interval neutrosophic number aggregation operators are developed. Then, a method for multicriteria decision making problems is explored applying the aggregation operators. In addition, an example is provided to illustrate the application of the proposed method.

## 1. Introduction

Zadeh proposed his remarkable theory of fuzzy sets (FSs in short) in 1965 [1] to encounter different types of uncertainties. Since then, it has been applied successfully in various fields [2]. As the traditional fuzzy set uses one single value *μ*_*A*_(*x*)∈[0,1] to represent the grade of membership of the fuzzy set *A* defined on a universe, it cannot handle some cases where *μ*_*A*_ is hard to be defined by a specific value. So interval valued fuzzy sets (IVFSs) were introduced by Turksen [3]. And to cope with the lack of knowledge of nonmembership degrees, Atanassov introduced intuitionistic fuzzy sets (IFSs in short) [4–7], an extension of Zadeh's FSs. In addition, Gau and Buehrer [8] defined vague sets. Later on, Bustince pointed out that vague sets and Atanassov's IFSs are mathematically equivalent objects [9]. As for the present, IFSs have been widely applied in solving multicriteria decision making problems [10–14], neural networks [15, 16], medical diagnosis [17], color region extraction [18, 19], market prediction [20], and so forth.

IFSs took into account the membership degree, nonmembership degree, and degree of hesitation simultaneously. So IFSs are more flexible and practical in addressing the fuzziness and uncertainty than the traditional FSs. Moreover, in some actual cases, the membership degree, nonmembership degree, and hesitation degree of an element in the IFS may not be a specific number. Hence, it was extended to the interval valued intuitionistic fuzzy sets (IVIFSs in brief) [21]. To handle the situations where people are hesitant in expressing their preference over objects in a decision making process, hesitant fuzzy sets (HFSs) were introduced by Torra [22] and Torra and Narukawa [23].

Although the FSs theory has been developed and generalized, it can not deal with all sorts of uncertainties in different real physical problems. Some types of uncertainties such as the indeterminate information and inconsistent information can not be handled. For example [24], when we ask about the opinion of an expert about a certain statement, he or she may say that the possibility that the statement is true is 0.5, that the statement is false is 0.6, and the degree that he or she is not sure is 0.2. This issue is beyond the scope of FSs and IFSs. Therefore, some new theories are required.

Smarandache coined neutrosophic logic and neutrosophic sets (NSs) in 1995 [25, 26]. A NS is a set where each element of the universe has a degree of truth, indeterminacy, and falsity, respectively, and which lies in ]0^−^, 1^+^[, the nonstandard unit interval [27]. Obviously, it is the extension to the standard interval [0,1] in IFSs. And the uncertainty present here, that is, indeterminacy factor, is independent of truth and falsity values while the incorporated uncertainty is dependent on the degrees of belongingness and nonbelongingness in IFSs [28]. And for the aforementioned example, by means of NSs, it can be expressed as *x*(0.5, 0.2, 0.6).

However, without being specified, it is difficult to apply in the real applications. Hence, the single valued neutrosophic set (SVNS) was proposed, which is an instance of NSs [24, 28]. Furthermore, the information energy of SVNSs, correlation and correlation coefficient of SVNSs, and a decision making method by the use of SVNSs were presented [29]. In addition, Ye also introduced the concept of simplified neutrosophic sets (SNSs), which can be described by three real numbers in the real unit interval [0,1], and proposed a multicriteria decision making method using aggregation operators for SNSs [30]. Majumdar and Samant introduced a measure of entropy of a SVNS [28].

In fact, sometimes the degree of truth, falsity, and indeterminacy of a certain statement can not be defined exactly in the real situations but denoted by several possible interval values. So the interval neutrosophic set (INS) was required, similar to IVIFS. Wang et al. proposed the concept of INS and gave the set-theoretic operators of INS [31]. The operations of INS were discussed in [32]; yet the comparison methods were not seen there. Furthermore, Ye defined the Hamming and Euclidean distances between INSs and proposed the similarity measures between INSs based on the relationship between similarity measures and distances [33]. However, in some cases, the INS operations in [31] might be irrational. For instance, the sum of any element and the maximum value should be equal to the maximum one, while it does not hold with the operations in [31]. In addition, to the best of our knowledge, the existing literatures do not put forward the INS aggregation operators and decision making method, which were vitally important for INSs to be utilized in the real situations in scientific and engineering applications. Therefore, the operations and comparison approach between interval neutrosophic numbers (INNs) and the aggregation operators for INSs are defined in this paper to be used. Thus, a multicriteria decision making method is established based on the proposed operators; an illustrative example is given to demonstrate the application of the proposed method.

The rest of the paper is organized as follows.  Section 2 briefly introduces interval numbers, properties of *t*-norm and *t*-conorm, and concepts and operations of NSs, SNSs, and INSs. And the operations and comparison approach for INSs are defined on the basis of the IVIFS theory in Section 3. The INN aggregation operators are given and a decision making method is developed for INSs by means of the INN aggregation operators in Section 4. In Section 5, an illustrative example is presented to illustrate the proposed method and the comparison analysis and discussion are given. Finally, Section 6 concludes the paper.

## 2. Preliminaries

In this section, some basic concepts and definitions related to INSs, including interval numbers, *t*-norm and *t-*conorm, and the definitions and operations of NSs, SNSs, and INSs are introduced, which will be utilized in the rest of the paper.

### 2.1. Interval Numbers and Their Operations

Interval numbers and their operations are of utmost importance to explore the operations for INSs. So some definitions and operations of interval numbers are given below.

**Definition 1 (see [34–37])**

Let $\tilde{a}=[a^{L},a^{U}]=\{x|a^{L}\leq x\leq a^{U}\}$, and then $\tilde{a}$ is called an interval number. In particular, if 0 ≤ *a* ^*L*^ ≤ *x* ≤ *a* ^*U*^, then $\tilde{a}$ is reduced to a positive interval number.

Consider any two interval numbers $\tilde{a}=[a^{L},a^{U}]$ and $\tilde{b}=[b^{L},b^{U}]$, and then their operations are defined as follows:

1. $\tilde{a}=\tilde{b}\Leftrightarrow a^{L}=b^{L},\;a^{U}=b^{U}$;
2. $\tilde{a}+\tilde{b}=[a^{L}+b^{L},a^{U}+b^{U}]$;
3. $\tilde{a}-\tilde{b}=[a^{L}-b^{U},a^{U}-b^{L}]$;
4. $\tilde{a}\times \tilde{b}=[\min\{a^{L}b^{L},a^{L}b^{U},a^{U}b^{L},a^{U}b^{U}\}$, max⁡{*a* ^*L*^ *b* ^*L*^, *a* ^*L*^ *b* ^*U*^, *a* ^*U*^ *b* ^*L*^, *a* ^*U*^ *b* ^*U*^}];
5. $k\tilde{a}=[ka^{L},ka^{U}],\;k>0$.

**Definition 2 (see [37])**

Let $\tilde{a}=[a^{L},a^{U}]$ and $\tilde{b}=[b^{L},b^{U}]$, $\;l_{\tilde{a}}={\tilde{a}}^{U}-{\tilde{a}}^{L}$ and $l_{\tilde{b}}={\tilde{b}}^{U}-{\tilde{b}}^{L}$, and then the degree of possibility of $\tilde{a}\geq \tilde{b}$ is formulated by

(1) $$\begin{array}{c} p\left(\tilde{a}\geq \tilde{b}\right)=\max\left\{1-\max\left(\frac{{\tilde{b}}^{U}-{\tilde{a}}^{L}}{l_{\tilde{a}}+l_{\tilde{b}}},0\right),0\right\}. \end{array}$$

Suppose that there are *n* interval numbers ${\tilde{a}}_{i}=[a_{i}^{L},a_{i}^{U}]\;(i=1,2,\ldots ,n)$ and each interval number ${\tilde{a}}_{i}$ is compared to all interval numbers ${\tilde{a}}_{j}\;(j=1,2,\ldots ,n)$ by using (1), namely,

(2) $$\begin{array}{c} p_{ij}=p\left({\tilde{a}}_{i}\geq {\tilde{a}}_{j}\right)=\max\left\{1-\max\left(\frac{{\tilde{a}}_{j}^{U}-{\tilde{a}}_{i}^{L}}{l_{{\tilde{a}}_{i}}+l_{{\tilde{a}}_{j}}},0\right),0\right\}. \end{array}$$

Then a complementary matrix can be constructed as follows:

(3) $$\begin{array}{c} P=\left[\begin{array}{cccc} p_{11} & p_{12} & \cdots & p_{1n}\\ p_{21} & p_{22} & \cdots & p_{2n}\\ & & \vdots & \\ p_{n1} & p_{n2} & \cdots & p_{nn} \end{array}\right], \end{array}$$

where *p*_*ij*_ ≥ 0, *p*_*ij*_ + *p*_*ji*_ = 1, *p*_*ii*_ = 0.5.

### 2.2. *t*-Norm and *t*-Conorm

The *t*-norm and its dual *t*-conorm play an important role in the construction of operation rules and averaging operators of INSs. Here, some basic concepts are introduced.

**Definition 3 (see [38, 39])**

A function *T* : [0,1]×[0,1]→[0,1] is called *t*-norm if it satisfies the following conditions:

1. ∀*x* ∈ [0,1],  *T*(1, *x*) = *x*;
2. ∀*x*, *y* ∈ [0,1],  *T*(*x*, *y*) = *T*(*y*, *x*);
3. ∀*x*, *y*, *z* ∈ [0,1],  *T*(*x*, *T*(*y*, *z*)) = *T*(*T*(*x*, *y*), *z*);
4. if *x* ≤ *x*′, *y* ≤ *y*′, then *T*(*x*, *y*) ≤ *T*(*x*′, *y*′).

**Definition 4 (see [38, 39])**

A function *S* : [0,1]×[0,1]→[0,1] is called *t*-conorm if it satisfies the following conditions:

1. ∀*x* ∈ [0,1],  *S*(0, *x*) = *x*;
2. ∀*x*, *y* ∈ [0,1],  *S*(*x*, *y*) = *S*(*y*, *x*);
3. ∀*x*, *y*, *z* ∈ [0,1],  *S*(*x*, *S*(*y*, *z*)) = *S*(*S*(*x*, *y*), *z*);
4. if *x* ≤ *x*′, *y* ≤ *y*′, then *S*(*x*, *y*) ≤ *S*(*x*′, *y*′).

**Definition 5 (see [38, 39])**

A *t*-norm function *T*(*x*, *y*) is called Archimedean *t*-norm if it is continuous and *T*(*x*, *x*) < *x* for all *x* ∈ (0,1). An Archimedean *t*-norm is called strictly Archimedean *t*-norm if it is strictly increasing in each variable for *x*, *y* ∈ (0,1). A *t*-conorm function *S*(*x*, *y*) is called Archimedean *t*-conorm if it is continuous and *S*(*x*, *x*) > *x* for all *x* ∈ (0,1). An Archimedean *t*-conorm is called strictly Archimedean *t*-conorm if it is strictly increasing in each variable for *x*, *y* ∈ (0,1).

It is well known [39, 40] that a strict Archimedean *t*-norm can be expressed via its additive generator *k* as *T*(*x*, *y*) = *k* ^−1^(*k*(*x*) + *k*(*y*)) and similarly applied to its dual *t*-conorm *S*(*x*, *y*) = *l* ^−1^(*l*(*x*) + *l*(*y*)) with *l*(*t*) = *k*(1 − *t*). We observe that an additive generator of a continuous Archimedean *t*-norm is a strictly decreasing function *k* : [0,1]→[0, *∞*).

There are some well-known Archimedean *t*-conorms and *t*-norms [41].

(1) Let *k*(*t*) = −log⁡*t*, *l*(*t*) = −log⁡(1 − *t*), *k* ^−1^(*t*) = *e* ^−*t*^, and *l* ^−1^(*t*) = 1 − *e* ^−*t*^. Then algebraic *t*-conorm and *t*-norm are obtained:
  (4) $$\begin{array}{c} S\left(x,y\right)=1-\left(1-x\right)\left(1-y\right),\;\;T\left(x,y\right)=xy. \end{array}$$
(2) Let *k*(*t*) = log⁡((2 − *t*)/*t*), *l*(*t*) = log⁡((2 − (1 − *t*))/(1 − *t*)), *k* ^−1^(*t*) = 2/(*e* ^*t*^ + 1), and *l* ^−1^(*t*) = 1 − (2/(*e* ^*t*^ + 1)). Then Einstein *t*-conorm and *t*-norm are obtained:
  (5) $$\begin{array}{c} S\left(x,y\right)=\frac{x+y}{1+xy},\;\;T\left(x,y\right)=\frac{xy}{1+\left(1-x\right)\left(1-y\right)}. \end{array}$$
(3) Let *k*(*t*) = log⁡((*γ* − (1 − *γ*)*t*)/*t*), *l*(*t*) = log⁡((*γ* − (1 − *γ*)(1 − *t*))/(1 − *t*)), *k* ^−1^(*t*) = *γ*/(*e* ^*t*^ + *γ* − 1), and *l* ^−1^(*t*) = 1 − (*γ*/(*e* ^*t*^ + *γ* − 1)), *γ* > 0. Then Hamacher *t*-conorm and *t*-norm are obtained:
  (6) $$\begin{array}{c} S\left(x,y\right)=\frac{x+y-xy-\left(1-\gamma \right)xy}{1-\left(1-\gamma \right)xy},\\ T\left(x,y\right)=\frac{xy}{\gamma +\left(1-\gamma \right)\left(x+y-xy\right)},\;\gamma >0. \end{array}$$

### 2.3. Definitions and Operations of NSs and SNSs

**Definition 6 (see [31])**

Let *X* be a space of points (objects), with a generic element in *X* denoted by *x*. A NS *A* in *X* is characterized by a truth-membership function *T*_*A*_(*x*), an indeterminacy-membership function *I*_*A*_(*x*), and a falsity-membership function *F*_*A*_(*x*).  *T*_*A*_(*x*), *I*_*A*_(*x*), and *F*_*A*_(*x*) are real standard or nonstandard subsets of ]0^−^, 1^+^[; that is, *T*_*A*_(*x*) : *X*→]0^−^, 1^+^[, *I*_*A*_(*x*) : *X*→]0^−^, 1^+^[, and *F*_*A*_(*x*) : *X*→]0^−^, 1^+^[. There is no restriction on the sum of *T*_*A*_(*x*), *I*_*A*_(*x*), and *F*_*A*_(*x*), so 0^−^ ≤ sup⁡*T*_*A*_(*x*) + sup⁡*I*_*A*_(*x*) + sup⁡*F*_*A*_(*x*) ≤ 3^+^.

**Definition 7 (see [31])**

A NS *A* is contained in the other NS *B*, denoted by *A*⊆*B*, if and only if inf⁡*T*_*A*_(*x*) ≤ inf⁡*T*_*B*_(*x*), sup⁡*T*_*A*_(*x*) ≤ sup⁡*T*_*B*_(*x*), inf⁡*I*_*A*_(*x*) ≤ inf⁡*I*_*B*_(*x*), sup⁡*I*_*A*_(*x*) ≤ sup⁡*I*_*B*_(*x*), inf⁡*F*_*A*_(*x*) ≤ inf⁡*F*_*B*_(*x*), and sup⁡*F*_*A*_(*x*) ≤ sup⁡*F*_*B*_(*x*) for *x* ∈ *X*.

Since it is difficult to apply NSs to practical problems, Ye reduced NSs of nonstandard intervals into a kind of SNSs of standard intervals that will preserve the operations of NSs [30].

**Definition 8 (see [30])**

Let *X* be a space of points (objects), with a generic element in *X* denoted by *x*. A NS *A* in *X* is characterized by *T*_*A*_(*x*), *I*_*A*_(*x*), and *F*_*A*_(*x*), which are single subintervals/subsets in the real standard [0, 1]; that is, *T*_*A*_(*x*) : *X* → [0,1], *I*_*A*_(*x*) : *X* → [0,1], and *F*_*A*_(*x*) : *X* → [0,1]. Then, a simplification of *A* is denoted by

(7) $$\begin{array}{c} A=\left\{\langle x,T_{A}\left(x\right),I_{A}\left(x\right),F_{A}\left(x\right)\rangle \;|\;x\in X\right\}, \end{array}$$

which is called a SNS. It is a subclass of NSs.

The operational relations of SNSs are also defined in [30].

**Definition 9 (see [30])**

Let *A* and *B* be two SNSs. For any *x* ∈ *X*,

1. *A* + *B* = 〈*T*_*A*_(*x*) + *T*_*B*_(*x*) − *T*_*A*_(*x*) · *T*_*B*_(*x*), *I*_*A*_(*x*) + *I*_*B*_(*x*) − *I*_*A*_(*x*) · *I*_*B*_(*x*),  *F*_*A*_(*x*) + *F*_*B*_(*x*) − *F*_*A*_(*x*) · *F*_*B*_(*x*)〉,
2. *A* · *B* = 〈*T*_*A*_(*x*) · *T*_*B*_(*x*), *I*_*A*_(*x*) · *I*_*B*_(*x*), *F*_*A*_(*x*) · *F*_*B*_(*x*)〉,
3. *λ* · *A* = 〈1 − (1 − *T*_*A*_(*x*))^*λ*^, 1 − (1 − *I*_*A*_(*x*))^*λ*^, 1 − (1 − *F*_*A*_(*x*))^*λ*^〉,  *λ* > 0,
4. *A* ^*λ*^ = 〈*T*_*A*_^*λ*^(*x*), *I*_*A*_^*λ*^(*x*), *F*_*A*_^*λ*^(*x*)〉,  *λ* > 0.

There are some limitations in Definition 9.

(1) In some situations, the operations, such as *A* + *B* and *A* · *B*, as given in Definition 9, might be irrational. This will be shown in the example below.

For example, let two simplified neutrosophic numbers (SNNs) *a* = 〈0.5,0.5,0.5〉 and *b* = 〈1,0, 0〉. Obviously, *b* = 〈1,0, 0〉 is the maximum of SNSs. It is notable that the sum of any number and the maximum number should be equal to the maximum one. However, according to (1) in Definition 9, *a* + *b* = 〈1,0.5,0.5〉 ≠ *b*. Hence, (1) does not hold and so do the other equations in Definition 9. It shows that the operations above are incorrect.

(2) In addition, the similarity measure for SNSs in [30] on the basis of the operations does not satisfy any cases.

For instance, let the alternatives *a*_1_ = 〈0.1,0, 0〉, *a*_2_ = 〈0.9,0, 0〉 and the ideal alternative a* = 〈1,0, 0〉. According to the decision making method based on the cosine similarity measure for SNSs under the simplified neutrosophic environment in [30], we can obtain that *S*_1_(*a*_1_, a*) = 1, *S*_2_(*a*_2_, a*) = 1; that is, the alternative *a*_1_ is equal to the alternative *a*_2_. However, for *T*_*a*_2__(*x*) > *T*_*a*_1__(*x*),   *I*_*a*_2__(*x*) > *I*_*a*_1__(*x*),   and *F*_*a*_2__(*x*) > *F*_*a*_1__(*x*), it is clear that the alternative *a*_2_ is superior to the alternative *a*_1_.

### 2.4. Definitions and Operations of INSs

**Definition 10 (see [31])**

Let *X* be a space of points (objects) with generic elements in *X* denoted by *X*. An INS *A* in *X* is characterized by a truth-membership function *T*_*A*_(*x*), an indeterminacy-membership function *I*_*A*_(*x*), and a falsity-membership function *F*_*A*_(*x*). For each point *x* in *X*, we have that *T*_*A*_(*x*) = [inf⁡*T*_*A*_(*x*), sup⁡*T*_*A*_(*x*)], *I*_*A*_(*x*) = [inf⁡*I*_*A*_(*x*), sup⁡*I*_*A*_(*x*)], *F*_*A*_(*x*) = [inf⁡*F*_*A*_(*x*), sup⁡*F*_*A*_(*x*)]⊆[0,1], and 0 ≤ sup⁡*T*_*A*_(*x*) + sup⁡*I*_*A*_(*x*) + sup⁡*F*_*A*_(*x*) ≤ 3, *x* ∈ *X*. We only consider the subunitary interval of [0, 1]. It is the subclass of a NS. Therefore, all INSs are clearly NSs.

**Definition 11 (see [31])**

An INS *A* is contained in the other INS *B*, *A*⊆*B*, if and only if

 inf⁡*T*_*A*_(*x*) ≤ inf⁡*T*_*B*_(*x*), sup⁡*T*_*A*_(*x*) ≤ sup⁡*T*_*B*_(*x*), inf⁡*I*_*A*_(*x*) ≥ inf⁡*I*_*B*_(*x*), sup⁡*I*_*A*_(*x*) ≥ sup⁡*I*_*B*_(*x*),
 inf⁡*F*_*A*_(*x*) ≥ inf⁡*F*_*B*_(*x*) and sup⁡*F*_*A*_(*x*) ≥ sup⁡*F*_*B*_(*x*),

for any *x* ∈ *X*.

**Definition 12 (see [31])**

Two INSs *A* and *B* are equal, written as *A* = *B*, if and only if *A*⊆*B* and *A*⊇*B*.

**Definition 13 (see [31])**

The addition of two INSs *A* and *B* is an INS *C*, written as *C* = *A* + *B*, whose truth-membership, indeterminacy-membership, and falsity-membership functions are related to those of *A* and *B* by

 inf⁡*T*_*C*_ = min⁡(inf⁡*T*_*A*_ + inf⁡*T*_*B*_, 1), sup⁡*T*_*C*_ = min⁡(sup⁡*T*_*A*_ + sup⁡*T*_*B*_, 1),
 inf⁡*I*_*C*_ = min⁡(inf⁡*I*_*A*_ + inf⁡*I*_*B*_, 1),   sup⁡*I*_*C*_ = min⁡(sup⁡*I*_*A*_ + sup⁡*I*_*B*_, 1),
 inf⁡*F*_*C*_ = min⁡(inf⁡*F*_*A*_ + inf⁡*F*_*B*_, 1), sup⁡*F*_*C*_ = min⁡(sup⁡*F*_*A*_ + sup⁡*F*_*B*_, 1),

for all *x* in *X*.

As to be known, when *B* = 〈0,1, 1〉, it should satisfy *A* + *B* = *A* and *A* · *B* = *B* for *B* being the minimum value of INSs. And when *B* = 〈1,0, 0〉, as the largest element of INSs, it should satisfy *A* + *B* = *B* and *A* + *B* = *A*. Let *B* = 〈1,0, 0〉. That is inf⁡*T*_*B*_ = sup⁡*T*_*B*_ = 1, inf⁡*I*_*B*_ = sup⁡*I*_*B*_ = 0, and inf⁡*F*_*B*_ = sup⁡*F*_*B*_ = 0. According to Definition 13, inf⁡*T*_*C*_ = 1, sup⁡*T*_*C*_ = 1, inf⁡*I*_*C*_ = inf⁡*I*_*A*_, sup⁡*I*_*C*_ = sup⁡*I*_*A*_, inf⁡*F*_*C*_ = inf⁡*F*_*A*_, and sup⁡*F*_*C*_ = sup⁡*F*_*A*_; that is, *A* + *B* = 〈[1,1], [inf⁡*I*_*A*_, sup⁡*I*_*A*_], [inf⁡*F*_*A*_, sup⁡*F*_*A*_]〉 ≠ *B*, so that Definition 13 does not hold.

**Definition 14 (see [31])**

The Cartesian product of two INSs *A* defined on the universe *X*_1_ and *B* defined on the universe *X*_2_ is an INS *C*, written as *C* = *A* · *B*, whose truth-membership, indeterminacy-membership, and falsity-membership functions are related to those of *A* and *B* by

 inf⁡*T*_*C*_(*x*, *y*) = inf⁡*T*_*A*_(*x*) + inf⁡*T*_*B*_(*y*) − inf⁡*T*_*A*_(*x*) · inf⁡*T*_*B*_(*y*),
 sup⁡*T*_*C*_(*x*, *y*) = sup⁡*T*_*A*_(*x*) + sup⁡*T*_*B*_(*y*) − sup⁡*T*_*A*_(*x*) · sup⁡*T*_*B*_(*y*),
 inf⁡*I*_*C*_(*x*, *y*) = inf⁡*I*_*A*_(*x*) · inf⁡*I*_*B*_(*y*), sup⁡*I*_*C*_(*x*, *y*) = sup⁡*I*_*A*_(*x*) · sup⁡*I*_*B*_(*y*),
 inf⁡*F*_*C*_(*x*, *y*) = inf⁡*F*_*A*_(*x*) · inf⁡*F*_*B*_(*y*), sup⁡*F*_*C*_(*x*, *y*) = sup⁡*F*_*A*_(*x*) · sup⁡*F*_*B*_(*y*),

for all *x* in *X*_1_, *y* in *X*_2_.

Being similar to Definition 13, Definition 14 does not hold in some cases. Therefore, new operation rules for INSs should be explored.

## 3. Operations and Comparison Approach for INSs

### 3.1. Operations for INSs

Xu defined some operations of interval valued intuitionistic fuzzy numbers [42]. Based on these operations and preliminaries in Section 2, the operations of two INSs can be defined as follows.

**Definition 15**

Let two INNs *A* = 〈[inf⁡*T*_*A*_, sup⁡*T*_*A*_], [inf⁡*I*_*A*_, sup⁡*I*_*A*_], [inf⁡*F*_*A*_, sup⁡*F*_*A*_]〉, *B* = 〈[inf⁡*T*_*B*_, sup⁡*T*_*B*_], [inf⁡*I*_*B*_, sup⁡*I*_*B*_], [inf⁡*F*_*B*_, sup⁡*F*_*B*_]〉, and *λ* > 0. The operations for INNs are defined based on the Archimedean *t*-conorm and *t*-norm as below:

(1) (8) λA=〈[l−1(λl(inf⁡TA)),l−1(λl(sup⁡TA))],[k−1(λk(inf⁡IA)),k−1(λk(sup⁡IA))],[k−1(λk(inf⁡FA)),k−1(λk(sup⁡FA))]〉;
(2) (9) Aλ=〈[(k−1(λk(inf⁡TA))),(k−1(λk(sup⁡TA)))],[l−1(λl(inf⁡IA)),l−1(λl(sup⁡IA))],[l−1(λl(inf⁡FA)),l−1(λl(sup⁡FA))]〉;
(3) (10) A+B =〈[l−1(l(inf⁡TA)+l(inf⁡TB)),l−1(l(sup⁡TA)+l(sup⁡TB))],  [k−1(k(inf⁡IA)+k(inf⁡IB)),  k−1(k(sup⁡IA)+k(sup⁡IB))],  [k−1(k(inf⁡FA)+k(inf⁡FB)),  k−1(k(sup⁡FA)+k(sup⁡FB))]〉;
(4) (11) A·B =〈[k−1(k(inf⁡TA)+k(inf⁡TB)),  k−1(k(sup⁡TA)+k(sup⁡TB))],  [l−1(l(inf⁡IA)+l(inf⁡IB)),  l−1(l(sup⁡IA)+l(sup⁡IB))],  [l−1(l(inf⁡FA)+l(inf⁡FB)),  l−1(l(sup⁡FA)+l(sup⁡FB))]〉.

Let *A* and *B* be both INNs. If we assign its generator *k* a specific form, specific operations for INSs will be obtained. When *k*(*x*) = −log⁡(*x*), we have

(5) (12) λA=〈[1−(1−inf⁡TA)λ,1−(1−sup⁡TA)λ],[(inf⁡IA)λ,(sup⁡IA)λ],[(inf⁡FA)λ,(sup⁡FA)λ]〉;
(6) (13) Aλ=〈[(inf⁡TA)λ,(sup⁡TA)λ],[1−(1−inf⁡IA)λ,1−(1−sup⁡IA)λ],[1−(1−inf⁡FA)λ,1−(1−sup⁡FA)λ]〉;
(7) (14) A+B=〈[inf⁡TA+inf⁡TB−inf⁡TA·inf⁡TB,sup⁡TA+sup⁡TB−sup⁡TA·sup⁡TB],[inf⁡TA·inf⁡IB,sup⁡IA·sup⁡IB],[inf⁡FA·inf⁡FB,sup⁡FA·sup⁡FB]〉;
(8) (15) A·B=〈[inf⁡TA·inf⁡TB,sup⁡TA·sup⁡TB],[inf⁡TA+inf⁡IB−inf⁡TA·inf⁡IB,sup⁡IA+sup⁡IB−sup⁡IA·sup⁡IB],[inf⁡FA+inf⁡FB−inf⁡FA·inf⁡FB,sup⁡FA+sup⁡FB−sup⁡FA·sup⁡FB]〉.

**Theorem 16**

Let three INNs *A* = 〈[inf⁡*T*_*A*_, sup⁡*T*_*A*_], [inf⁡*I*_*A*_, sup⁡*I*_*A*_], [inf⁡*F*_*A*_, sup⁡*F*_*A*_]〉, *B* = 〈[inf⁡*T*_*B*_, sup⁡*T*_*B*_], [inf⁡*I*_*B*_, sup⁡*I*_*B*_], [inf⁡*F*_*B*_, sup⁡*F*_*B*_]〉, *C* = 〈[inf⁡*T*_*C*_, sup⁡*T*_*C*_], [inf⁡*I*_*C*_, sup⁡*I*_*C*_], [inf⁡*F*_*C*_, sup⁡*F*_*C*_]〉, and then the following equations are true:

1. *A* + *B* = *B* + *A*,
2. *A* · *B* = *B* · *A*,
3. *λ*(*A* + *B*) = *λA* + *λB*,  *λ* > 0,
4. (*A* · *B*)^*λ*^ = *A* ^*λ*^ + *B* ^*λ*^,  *λ* > 0,
5. *λ*_1_ *A* + *λ*_2_ *A* = (*λ*_1_ + *λ*_2_)*A*,  *λ*_1_ > 0,  *λ*_2_ > 0,
6. *A* ^*λ*_1_^ · *A* ^*λ*_2_^ = *A* ^(*λ*_1_+*λ*_2_)^,  *λ*_1_ > 0,  *λ*_2_ > 0,
7. (*A* + *B*) + *C* = *A* + (*B* + *C*),
8. (*A* · *B*) · *C* = *A* · (*B* · *C*).

**Proof**

(1), (2), (7), and (8) are obvious; thus we prove the others:

(3) (16) λ(A+B)=λ·〈[l−1(l(inf⁡TA)+l(inf⁡TB)),l−1(l(sup⁡TA)+l(sup⁡TB))],[k−1(k(inf⁡IA)+k(inf⁡IB)),  k−1(k(sup⁡IA)+k(sup⁡IB))],[k−1(k(inf⁡FA)+k(inf⁡FB)),k−1(k(sup⁡FA)+k(sup⁡FB))]〉=〈[l−1(λl(l−1(l(inf⁡TA)+l(inf⁡TB)))),l−1(λl(l−1(l(sup⁡TA)+l(sup⁡TB))))],[k−1(λk(k−1(k(inf⁡IA)+k(inf⁡IB)))),k−1(λk(k−1(k(sup⁡IA)+k(sup⁡IB))))],[k−1(λk(k−1(k(inf⁡FA)+k(inf⁡FB)))),k−1(λk(k−1(k(sup⁡FA)+k(sup⁡FB))))]〉=〈[l−1(λ(l(inf⁡TA)+l(inf⁡TB))),l−1(λ(l(sup⁡TA)+l(sup⁡TB)))],[k−1(λ(k(inf⁡IA)+k(inf⁡IB))),k−1(λ(k(sup⁡IA)+k(sup⁡IB)))],[k−1(λ(k(inf⁡FA)+k(inf⁡FB))),k−1(λ(k(sup⁡FA)+k(sup⁡FB)))]〉=〈[l−1(λl(inf⁡TA)+λl(inf⁡TB)),l−1(λl(sup⁡TA)+λl(sup⁡TB))],[k−1(λk(inf⁡IA)+λk(inf⁡IB)),k−1(λk(sup⁡IA)+λk(sup⁡IB))],[k−1(λk(inf⁡FA)+λk(inf⁡FB)),k−1(λk(sup⁡FA)+λk(sup⁡FB))]〉=λA+λB
(4) (17) (A·B)λ=(〈[k−1(k(inf⁡TA)+k(inf⁡TB)),k−1(k(sup⁡TA)+k(sup⁡TB))],[l−1(l(inf⁡IA)+l(inf⁡IB)),l−1(l(sup⁡IA)+l(sup⁡IB))],[l−1(l(inf⁡FA)+l(inf⁡FB)),l−1(l(sup⁡FA)+l(sup⁡FB))]〉)λ=〈[k−1(λk(k−1(k(inf⁡TA)+k(inf⁡TB)))),k−1(λk(k−1(k(sup⁡TA)+k(sup⁡TB))))],[l−1(λl(l−1(l(inf⁡IA)+l(inf⁡IB)))),l−1(λl(l−1(l(sup⁡IA)+l(sup⁡IB))))],[l−1(λl(l−1(l(inf⁡FA)+l(inf⁡FB)))),l−1(λl(l−1(l(sup⁡FA)+l(sup⁡FB))))]〉=〈[k−1(λ(k(inf⁡TA)+k(inf⁡TB))),k−1(λ(k(sup⁡TA)+k(sup⁡TB)))],[l−1(λ(l(inf⁡IA)+l(inf⁡IB))),l−1(λ(l(sup⁡IA)+l(sup⁡IB)))],[l−1(λ(l(inf⁡FA)+l(inf⁡FB))),l−1(λ(l(sup⁡FA)+l(sup⁡FB)))]〉=〈[k−1(λk(inf⁡TA)+λk(inf⁡TB)),k−1(λk(sup⁡TA)+λk(sup⁡TB))],[l−1(λl(inf⁡IA)+λl(inf⁡IB)),l−1(λl(sup⁡IA)+λl(sup⁡IB))],[l−1(λl(inf⁡FA)+λl(inf⁡FB)),l−1(λl(sup⁡FA)+λl(sup⁡FB))]〉=Aλ·Bλ
(5) (18) λ1A+λ2A =〈[l−1(λ1l(inf⁡TA)),l−1(λ1l(sup⁡TA))],  [k−1(λ1k(inf⁡IA)),k−1(λ1k(sup⁡IA))],  [k−1(λ1k(inf⁡FA)),k−1(λ1k(sup⁡FA))]〉  ⊕〈[l−1(λ2l(inf⁡TA)),l−1(λ2l(sup⁡TA))],  [k−1(λ2k(inf⁡IA)),k−1(λ2k(sup⁡IA))],  [k−1(λ2k(inf⁡FA)),k−1(λ2k(sup⁡FA))]〉 =〈[l−1(l(l−1(λ1l(inf⁡TA)))+l(l−1(λ2l(inf⁡TA)))),  l−1(l(l−1(λ1l(sup⁡TA)))+l(l−1(λ2l(sup⁡TA))))],  [k−1(k(k−1(λ1k(inf⁡IA)))  +k(k−1(λ2k(inf⁡IA)))),  k−1(k(k−1(λ1k(sup⁡IA)))  +k(k−1(λ2k(sup⁡IA))))],  [k−1(k(k−1(λ1k(inf⁡FA)))  +k(k−1(λ2k(inf⁡FA)))),  k−1(k(k−1(λ1k(sup⁡FA)))  +k(k−1(λ2k(sup⁡FA))))]〉 =〈[l−1(λ1l(inf⁡TA)+λ2l(inf⁡TA)),  l−1(λ1l(sup⁡TA)+λ2l(sup⁡TA))],  [k−1(λ1k(inf⁡IA)+λ2k(inf⁡IA)),  k−1(λ1k(sup⁡IA)+λ2k(sup⁡IA))],  [k−1(λ1k(inf⁡FA)+λ2k(inf⁡FA)),  k−1(λ1k(sup⁡FA)+λ2k(sup⁡FA))]〉 =〈[l−1((λ1+λ2)l(inf⁡TA)),  l−1((λ1+λ2)l(sup⁡TA))],  [k−1((λ1+λ2)k(inf⁡IA)),  k−1((λ1+λ2)k(sup⁡IA))],  [k−1((λ1+λ2)k(inf⁡FA)),  k−1((λ1+λ2)k(sup⁡FA))]〉 =(λ1+λ2)A
(6) (19) Aλ1·Aλ2 =〈[k−1(λ1k(inf⁡TA)),k−1(λ1k(sup⁡TA))], [l−1(λ1l(inf⁡IA)),l−1(λ1l(sup⁡IA))], [l−1(λ1l(inf⁡FA)),l−1(λ1l(sup⁡FA))]〉  ·〈[k−1(λ2k(inf⁡TA)),k−1(λ2k(sup⁡TA))], [l−1(λ2l(inf⁡IA)),l−1(λ2l(sup⁡IA))], [l−1(λ2l(inf⁡FA)),l−1(λ2l(sup⁡FA))]〉 =〈[k−1(k(k−1(λ1k(inf⁡TA)))   +k(k−1(λ2k(inf⁡TA)))), k−1(k(k−1(λ1k(sup⁡TA))) +k(k−1(λ2k(sup⁡TA))))], [l−1(l(l−1(λ1l(inf⁡IA))) +l(l−1(λ2l(inf⁡IA)))), l−1(l(l−1(λ1l(sup⁡IA))) +l(l−1(λ2l(sup⁡IA))))], [l−1(l(l−1(λ1l(inf⁡FA))) +l(l−1(λ2l(inf⁡FA)))), l−1(l(l−1(λ1l(sup⁡FA))) +l(l−1(λ2l(sup⁡FA))))]〉 =〈[k−1(λ1k(inf⁡TA)+λ2k(inf⁡TA)), k−1(λ1k(sup⁡TA)+λ2k(sup⁡TA))], [l−1(λ1l(inf⁡IA)+λ2l(inf⁡IA)), l−1(λ1l(sup⁡IA)+λ2l(sup⁡IA))], [l−1(λ1l(inf⁡FA)+λ2l(inf⁡FA)), l−1(λ1l(sup⁡FA)+λ2l(sup⁡FA))]〉 =〈[k−1((λ1+λ2)k(inf⁡TA)), k−1((λ1+λ2)k(sup⁡TA))], [l−1((λ1+λ2)l(inf⁡IA)), l−1((λ1+λ2)l(sup⁡IA))], [l−1((λ1+λ2)l(inf⁡FA)), l−1((λ1+λ2)l(sup⁡FA))]〉 =Aλ1+λ2.

**Example 17**

Assume that *A* = 〈[0.7,0.8], [0.0,0.1], [0.1,0.2]〉, *B* = 〈[0.4,0.5], [0.2,0.3], [0.3,0.4]〉, and *λ* = 2. When *k*(*x*) = −log⁡(*x*), then

1. 2 · *A* = 〈[0.91,0.96], [0,0.01], [0.01,0.04]〉;
2. *A* ^2^ = 〈[0.49,0.64], [0,0.19], [0.19,0.36]〉;
3. *A* + *B* = 〈[0.82,0.90], [0,0.05], [0.03,0.08]〉;
4. *A* · *B* = 〈[0.28,0.40], [0.20,0.37], [0.37,0.52]〉.

INSs are the extension of SVNSs or SNSs. Assume that inf⁡*T*_*A*_(*x*) = sup⁡*T*_*A*_(*x*), inf⁡*I*_*A*_(*x*) = sup⁡*I*_*A*_(*x*), inf⁡*F*_*A*_(*x*) = sup⁡*F*_*A*_(*x*), inf⁡*T*_*B*_(*x*) = sup⁡*T*_*B*_(*x*), inf⁡*I*_*B*_(*x*) = sup⁡*I*_*B*_(*x*), and inf⁡*F*_*B*_(*x*) = sup⁡*F*_*B*_(*x*), and then the two INSs *A* = 〈*T*_*A*_(*x*), *I*_*A*_(*x*), *F*_*A*_(*x*)〉 and *B* = 〈*T*_*B*_(*x*), *I*_*B*_(*x*), *F*_*B*_(*x*)〉 are reduced to SNSs and SVNSs. According to Definition 15 and Theorem 16, the SNS or SVNS operations can be obtained.

IVIFSs are an instance of NSs. Let inf⁡*I*_*A*_ = sup⁡*I*_*A*_ = 0, inf⁡*I*_*B*_ = sup⁡*I*_*B*_ = 0, sup⁡*T*_*A*_ + sup⁡*F*_*A*_ ≤ 1, and sup⁡*T*_*B*_ + sup⁡*F*_*B*_ ≤ 1. Then the two INSs *A* = 〈*T*_*A*_, *I*_*A*_, *F*_*A*_〉 and *B* = 〈*T*_*B*_, *I*_*B*_, *F*_*B*_〉 are reduced to IVIFSs. According to Definition 15, when *k*(*x*) = −log⁡(*x*), the following equations can be obtained:

1. *A* + *B* = 〈[inf⁡*T*_*A*_ + inf⁡*T*_*B*_ − inf⁡*T*_*A*_ · inf⁡*T*_*B*_, sup⁡*T*_*A*_ + sup⁡*T*_*B*_ − sup⁡*T*_*A*_ · sup⁡*T*_*B*_],[inf⁡*F*_*A*_ · inf⁡*F*_*B*_, sup⁡*F*_*A*_ · sup⁡*F*_*B*_]〉,
2. *A* · *B* = 〈[inf⁡*T*_*A*_ · inf⁡*T*_*B*_, sup⁡*T*_*A*_ · sup⁡*T*_*B*_], [inf⁡*F*_*A*_ + inf⁡*F*_*B*_ − inf⁡*F*_*A*_ · inf⁡*F*_*B*_, sup⁡*F*_*A*_ + sup⁡*F*_*B*_ − sup⁡*F*_*A*_ · sup⁡*F*_*B*_]〉,
3. *λ* · *A* = 〈[1 − (1 − inf⁡*T*_*A*_)^*λ*^, 1 − (1 − sup⁡*T*_*A*_)^*λ*^], [(inf⁡*F*_*A*_)^*λ*^, (sup⁡*F*_*A*_)^*λ*^]〉,
4. *A* ^*λ*^ = 〈[(inf⁡*T*_*A*_)^*λ*^, (sup⁡*T*_*A*_)^*λ*^], [1 − (1 − inf⁡*F*_*A*_)^*λ*^, 1 − (1 − sup⁡*F*_*A*_)^*λ*^]〉,

which coincides with the operations of IVIFSs in [42]. It indicates that the same principles of INSs in Definition 15 also adapt to IVIFSs. In fact, when the indeterminacy factor *i* is replaced by *π* = 1 − *T* − *F*, the NS is an IFS.

### 3.2. Comparison Rules

Based on the score function and accuracy function of IVIFSs, the score function, accuracy function, and certainty function of an INN *A* are defined.

**Definition 18**

Let the INN *A* = 〈[inf⁡*T*_*A*_, sup⁡*T*_*A*_], [inf⁡*I*_*A*_, sup⁡*I*_*A*_], [inf⁡*F*_*A*_, sup⁡*F*_*A*_]〉, and then

1. *s*(*A*) = [inf⁡*T*_*A*_ + 1 − sup⁡*I*_*A*_ + 1 − sup⁡*F*_*A*_, sup⁡*T*_*A*_ + 1 − inf⁡*I*_*A*_ + 1 − inf⁡*F*_*A*_],
2. *a*(*A*) = [min⁡{inf⁡*T*_*A*_ − inf⁡*F*_*A*_, sup⁡*T*_*A*_ − sup⁡*F*_*A*_}, max⁡{inf⁡*T*_*A*_ − inf⁡*F*_*A*_, sup⁡*T*_*A*_ − sup⁡*F*_*A*_}],
3. *c*(*A*) = [inf⁡*T*_*A*_, sup⁡*T*_*A*_],

where *s*(*A*), *a*(*A*), and *c*(*A*) represent the score function, accuracy function, and certainty function of the INN *A*, respectively.

The score function is an important index in ranking INNs. For an INN *A*, the bigger the truth-membership *T*_*A*_ is, the greater the INS is. And the less the indeterminacy-membership *I*_*A*_ is, the greater the INS is. Similarly, the smaller the false-membership *F*_*A*_ is, the greater the INS is. At the same time, inf⁡*T*_*A*_(*x*), sup⁡*T*_*A*_(*x*), inf⁡*I*_*A*_(*x*), sup⁡*I*_*A*_(*x*), inf⁡*F*_*A*_(*x*), sup⁡*F*_*A*_(*x*)⊆[0,1], so the score function* s*(*A*) is defined as shown above. For the accuracy function, if the difference between truth and falsity is bigger, then the statement is surer. That is, the larger the values of *T*, *I,* and* F* are, the more the accuracy of the INS is. So the accuracy function is given above. As to the certainty function, the value of truth-membership *T*_*A*_ is bigger, and it means more certainty of the INS.

**Example 19**

Assume that *A* = 〈[0.7,0.8], [0.0,0.1], [0.1,0.2]〉, and *B* = 〈[0.4,0.5], [0.2,0.3], [0.3,0.4]〉, and then

1. *s*(*A*) = [2.4,2.7], *s*(*B*) = [1.7,2.0],
2. *a*(*A*) = [0.6,0.6], *a*(*B*) = [0.1,0.1],
3. *c*(*A*) = [0.7,0.8], *c*(*B*) = [0.4,0.5].

On the basis of Definition 18, the method to compare INNs can be defined as follows.

**Definition 20**

Let *A* and *B* be two INNs. The comparison approach can be defined as follows.

1. If *p*(*s*(*A*) ≥ *s*(*B*)) > 0.5, then *A* is greater than *B*; that is, *A* is superior to *B*, denoted by *A*≻*B*.
2. If *p*(*s*(*A*) ≥ *s*(*B*)) = 0.5 and *p*(*a*(*A*) ≥ *a*(*B*)) > 0.5, then *A* is greater than *B*; that is, *A* is superior to *B*, denoted by *A*≻*B*.
3. If *p*(*s*(*A*) ≥ *s*(*B*)) = 0.5, *p*(*a*(*A*) ≥ *a*(*B*)) = 0.5, and *p*(*c*(*A*) ≥ *c*(*B*)) > 0.5, then *A* is greater than *B*; that is, *A* is superior to *B*, denoted by *A*≻*B*.
4. If *p*(*s*(*A*) ≥ *s*(*B*)) = 0.5, *p*(*a*(*A*) ≥ *a*(*B*)) = 0.5, and *p*(*c*(*A*) ≥ *c*(*B*)) = 0.5, then *A* is equal to *B*; that is, *A* is indifferent to *B*, denoted by *A* ~ *B*.

**Example 21**

Let *A* and *B* be two INNs.

(1) Assume that *A* = 〈[0.7,0.8], [0.0,0.1], [0.1,0.2]〉 and *B* = 〈[0.4,0.5], [0.2,0.3], [0.3,0.4]〉. Referring to Definition 18, *s*(*A*) = [2.4,2.7], *s*(*B*) = [1.7,2.0], *a*(*A*) = [0.6,0.6], *a*(*B*) = [0.1,0.1], *c*(*A*) = [0.7,0.8], and *c*(*B*) = [0.4,0.5]. According to Definition 20, *p*(*s*(*A*) ≥ *s*(*B*)) = 1 > 0.5. Therefore, *A*≻*B*.

(2) Assuming that *A* = 〈[0.6,0.7], [0.3,0.4], [0.4,0.5]〉 and *B* = 〈[0.4,0.5], [0.2,0.3], [0.3,0.4]〉, referring to Definition 18, *s*(*A*) = [1.7,2.0], *s*(*B*) = [1.7,2.0], *a*(*A*) = [0.2,0.2], *a*(*B*) = [0.1,0.1], *c*(*A*) = [0.6,0.7], and *c*(*B*) = [0.4,0.5]. According to Definition 20, *p*(*s*(*A*) ≥ *s*(*B*)) = 0.5, *p*(*a*(*A*) ≥ *a*(*B*)) = 1 > 0.5. Therefore, *A*≻*B*.

(3) For two INNs *A* = 〈[0.6,0.7], [0.3,0.4], [0.4,0.5]〉 and *B* = 〈[0.4,0.5], [0.3,0.4], [0.2,0.3]〉, referring to Definition 18, *s*(*A*) = [1.7,2.0], *s*(*B*) = [1.7,2.0], *a*(*A*) = [0.2,0.2], *a*(*B*) = [0.2,0.2], *c*(*A*) = [0.6,0.7], and *c*(*B*) = [0.4,0.5]. According to Definition 20, *p*(*s*(*A*) ≥ *s*(*B*)) = 0.5, *p*(*a*(*A*) ≥ *a*(*B*)) = 0.5, and *p*(*c*(*A*) ≥ *c*(*B*)) = 1 > 0.5. Therefore, *A*≻*B*.

## 4. INN Aggregation Operators and Their Applications to Multicriteria Decision Making Problems

In this section, applying the INS operations, we present aggregation operators for INNs and propose a method for multicriteria decision making by means of the aggregation operators.

### 4.1. INN Aggregation Operators

**Definition 22**

Let *A*_*j*_ = 〈*T*_*A*_*j*__, *I*_*A*_*j*__, *F*_*A*_*j*__〉 (*j* = 1,2,…, *n*) be a collection of INNs, and let INNWA : INN^*n*^ → INN,

(20) INNWAw(A1,A2,…,An)=w1A1+w2A2+⋯+wnAn=∑j=1nwjAj;

then INNWA is called the interval neutrosophic number weighted averaging operator of dimension *n*, where *W* = (*w*_1_, *w*_2_,…, *w*_*n*_) is the weight vector of *A*_*j*_(*j* = 1,2,…, *n*), with *w*_*j*_ ≥ 0(*j* = 1,2,…, *n*) and ∑_*j*=1_^*n*^ *w*_*j*_ = 1.

**Theorem 23**

Let *A*_*j*_ = 〈*T*_*A*_*j*__, *I*_*A*_*j*__, *F*_*A*_*j*__〉  (*j* = 1,2,…, *n*) be a collection of INNs, and *W* = (*w*_1_, *w*_2_,…, *w*_*n*_) be the weight vector of *A*_*j*_  (*j* = 1,2,…, *n*), with *w*_*j*_ ≥ 0  (*j* = 1,2,…, *n*) and ∑_*j*=1_^*n*^ *w*_*j*_ = 1; then their aggregated result using the INNWA operator is also an INN, and

(21) INNWAw(A1,A2,…,An) =〈[l−1(w1l(inf⁡TA1)+w2l(inf⁡TA2)⋯+wnl(inf⁡TAn)),l−1(w1l(sup⁡TA1)+w2l(sup⁡TA2)⋯+wnl(sup⁡TAn))],[k−1(w1k(inf⁡IA1)+w2k(inf⁡IA2)⋯+wnk(inf⁡IAn)),k−1(w1k(sup⁡IA1)+w2k(sup⁡IA2)⋯+wnk(sup⁡IAn))],[k−1(w1k(inf⁡FA1)+w2k(inf⁡FA2)⋯+wnk(inf⁡FAn)),k−1(w1k(sup⁡FA1)+w2k(sup⁡FA2)⋯+wnk(sup⁡FAn))]〉,

where *k* is the additive generator of Archimedean *t*-norm that is used in the operations of INSs and *l*(*x*) = *k*(1 − *x*).

Let *k*(*x*) = −log⁡(*x*). Then *l*(*x*) = −log⁡(1 − *x*), *k* ^−1^(*x*) = *e* ^−*x*^, and *l* ^−1^(*x*) = 1 − *e* ^−*x*^. And the aggregated result using the INNWA operator in Theorem 23 can be represented by

(22) INNWAw(A1,A2,…,An) =〈[1−∏i=1n(1−inf⁡TAi)wi,1−∏i=1n(1−sup⁡TAi)wi],[∏i=1ninf⁡IAiwi,∏i=1nsup⁡IAiwi],[∏i=1ninf⁡FAiwi,∏i=1nsup⁡FAiwi]〉,

where *W* = (*w*_1_, *w*_2_, ..., *w*_*n*_) is the weight vector of *A*_*j*_  (*j* = 1,2,…, *n*), with *w*_*j*_ ∈ [0,1] and ∑_*j*=1_^*n*^ *w*_*j*_ = 1.

**Proof**

By using the mathematical induction on *n* we have the following.

(1) For *n* = 2, since

(23) w1A1+w2A2 =〈[l−1(w1l(inf⁡TA1)),l−1(w1l(sup⁡TA1))],[k−1(w1k(inf⁡IA1)),k−1(w1k(sup⁡IA1))],[k−1(w1k(inf⁡FA1)),k−1(w1k(sup⁡FA1))]〉  ⊕〈[l−1(w2l(inf⁡TA2)),l−1(w2l(sup⁡TA2))],[k−1(w2k(inf⁡IA2)),k−1(w2k(sup⁡IA2))],[k−1(w2k(inf⁡FA2)),k−1(w2k(sup⁡FA2))]〉 =〈[l−1(l(l−1(w1l(inf⁡TA1)))  +l(l−1(w2l(inf⁡TA2)))),l−1(l(l−1(w1l(sup⁡TA1)))+l(l−1(w2l(sup⁡TA2))))],  [k−1(k(k−1(w1k(inf⁡IA1)))+k(k−1(w2k(inf⁡IA2)))),k−1(k(k−1(w1k(sup⁡IA1)))+k(k−1(w2k(sup⁡IA2))))],  [k−1(k(k−1(w1k(inf⁡FA1)))+k(k−1(w2k(inf⁡FA2)))),k−1(k(k−1(w1k(sup⁡FA1)))  +k(k−1(w2k(sup⁡FA2))))]〉 =〈[l−1(w1l(inf⁡TA1)+w2l(inf⁡TA2)),  l−1(w1l(sup⁡TA1)+w2l(sup⁡TA2))],  [k−1(w1k(inf⁡IA1)+w2k(inf⁡IA2)),  k−1(w1k(sup⁡IA1)+w2k(sup⁡IA2))],  [k−1(w1k(inf⁡FA1)+w2k(inf⁡FA2)),  k−1(w1k(sup⁡FA1)+w2k(sup⁡FA2))]〉,

then

(24) SNNWAw(A1,A2) =w1A1+w2A2 =〈[l−1(w1l(inf⁡TA1)+w2l(inf⁡TA2)),l−1(w1l(sup⁡TA1)+w2l(sup⁡TA2))],[k−1(w1k(inf⁡IA1)+w2k(inf⁡IA2)),k−1(w1k(sup⁡IA1)+w2k(sup⁡IA2))],[k−1(w1k(inf⁡FA1)+w2k(inf⁡FA2)),k−1(w1k(sup⁡FA1)+w2k(sup⁡FA2))]〉.

(2) If (21) holds for *n* = *k*; that is,

(25) SNNWAw(A1,A2,…,Ak) =〈[l−1(∑j=1kwjl(inf⁡TAj)),l−1(∑j=1kwjl(sup⁡TAj))],[k−1(∑j=1kwjk(inf⁡IAj)),k−1(∑j=1kwjk(sup⁡IAj))],[k−1(∑j=1kwjk(inf⁡FAj)),k−1(∑j=1kwjk(sup⁡FAj))]〉,

then, if *n* = *k* + 1, we have

(26) SNNWAw(A1,A2,…,Ak,Ak+1) =〈[l−1(l(l−1(∑j=1kwjl(inf⁡TAj))+l(l−1(wk+1l(inf⁡TAk+1))))),l−1(l(l−1(∑j=1kwjl(sup⁡TAj))+l(l−1(wk+1l(sup⁡TAk+1)))))],[k−1(k(k−1(∑j=1kwjk(inf⁡IAj))+k(k−1(wk+1k(inf⁡IAk+1))))),k−1(k(k−1(∑j=1kwjk(sup⁡IAj))+k(k−1(wk+1k(sup⁡IAk+1)))))],[k−1(k(k−1(∑j=1kwjk(inf⁡FAj)))+k(k−1(wk+1k(inf⁡FAk+1)))),k−1(k(k−1(∑j=1kwjk(sup⁡FAj)))+k(k−1(wk+1k(sup⁡FAk+1))))]〉 =〈[l−1(∑j=1k+1wjl(inf⁡TAj)),l−1(∑j=1k+1wjl(sup⁡TAj))],[k−1(∑j=1k+1wjk(inf⁡IAj)),k−1(∑j=1k+1wjk(sup⁡IAj))],[k−1(∑j=1k+1wjk(inf⁡FAj)),k−1(∑j=1k+1wjk(sup⁡FAj))]〉;

that is, (29) holds for *n* = *k* + 1. Thus, (29) holds for all *n*. Then, we have

(27) SNNWAw(A1,A2,…,An) =〈[l−1(∑j=1nwjl(inf⁡TAj)),l−1(∑j=1nwjl(sup⁡TAj))],[k−1(∑j=1nwjk(inf⁡IAj)),k−1(∑j=1nwjk(sup⁡IAj))],[k−1(∑j=1nwjk(inf⁡FAj)),k−1(∑j=1nwjk(sup⁡FAj))]〉,

which completes the proof.

It is obvious that the INNWG operator has the following properties.

1. Idempotency: let *A*_*j*_ (*j* = 1,2,…, *n*) be a collection of INNs. If all *A*_*j*_ (*j* = 1,2,…, *n*) are equal, that is, *A*_*j*_ = *A*, for all *j* ∈ {1,2,…, *n*}, then INNWA_*w*_(*A*_1_, *A*_2_,…, *A*_*n*_) = *A*.
2. Boundedness: assume that *A*_*j*_ (*j* = 1,2,…, *n*) is a collection of INNs and *A* ^−^ = 〈min⁡_*j*_⁡*T*_*A*_*j*__(*x*), max⁡_*j*_ *I*_*A*_*j*__(*x*), max⁡_*j*_ *F*_*A*_*j*__(*x*)〉, *A* ^+^ = 〈max⁡_*j*_ *T*_*A*_*j*__(*x*), min⁡_*j*_ *I*_*A*_*j*__(*x*), min⁡_*j*_ *F*_*A*_*j*__(*x*)〉, for all *j* ∈ {1,2,…, *n*}, and then *A* ^−^⊆INNWA_*w*_(*A*_1_, *A*_2_,…, *A*_*n*_)⊆*A* ^+^.
3. Monotonity: assuming that *A*_*j*_ (*j* = 1,2,…, *n*) is a collection of INNs, if *A*_*j*_⊆*A*_j_*, for *j* ∈ {1,2,…, *n*}, then INNWA_*w*_(*A*_1_, *A*_2_,…, *A*_*n*_)⊆INNWA_*w*_(*A*_1_*, *A*_2_*,…, *A*_n_*).

**Definition 24**

Let *A*_*j*_ = 〈*T*_*A*_*j*__, *I*_*A*_*j*__, *F*_*A*_*j*__〉 (*j* = 1,2,…, *n*) be a collection of INNs, and let INNWG : INN^*n*^ → INN,

(28) $$\begin{array}{c} \text{INNW}{\text{G}}_{w}\left(A_{1},A_{2},\ldots ,A_{n}\right)=\prod_{j=1}^{n}A_{j}^{w_{i}}; \end{array}$$

then INNWG is called an interval neutrosophic number weighted geometric operator of dimension *n*, where *W* = (*w*_1_, *w*_2_,…, *w*_*n*_) is the weight vector of *A*_*j*_ (*j* = 1,2,…, *n*), with *w*_*j*_ ≥ 0 (*j* = 1,2,…, *n*) and ∑_*j*=1_^*n*^ *w*_*j*_ = 1.

**Theorem 25**

Let *A*_*j*_ = 〈*T*_*A*_*j*__, *I*_*A*_*j*__, *F*_*A*_*j*__〉  (*j* = 1,2,…, *n*) be a collection of INNs, and one has the following result by using Definition 15:

(29) INNWGw(A1,A2,…,An) =〈[k−1(w1k(inf⁡TA1)+w2k(inf⁡TA2)⋯+wnk(inf⁡TAn)),k−1(w1k(sup⁡TA1)+w2k(sup⁡TA2)⋯+wnk(sup⁡TAn))],[l−1(w1l(inf⁡IA1)+w2l(inf⁡IA2)⋯+wnl(inf⁡IAn)),l−1(w1l(sup⁡IA1)+w2l(sup⁡IA2)⋯+wnl(sup⁡IAn))],[l−1(w1l(inf⁡FA1)+w2l(inf⁡FA2)⋯+wnl(inf⁡FAn)),l−1(w1l(sup⁡FA1)+w2l(sup⁡FA2)⋯+wnl(sup⁡FAn))]〉.

Assume that *k*(*x*) = −log⁡(*x*), and then *l*(*x*) = −log⁡(1 − *x*), *k* ^−1^(*x*) = *e* ^−*x*^, *l* ^−1^(*x*) = 1 − *e* ^−*x*^. The aggregated result using the INNWG operator in Theorem 25 can be represented by

(30) INNWGw(A1,A2,…,An) =〈[∏i=1ninf⁡TAiwi,∏i=1nsup⁡TAiwi],[1−∏i=1n(1−inf⁡IAi)wi,1−∏i=1n(1−sup⁡IAi)wi],[1−∏i=1n(1−inf⁡FAi)wi,1−∏i=1n(1−sup⁡FAi)wi]〉,

where *W* = (*w*_1_, *w*_2_,…, *w*_*n*_) is the weight vector of *A*_*j*_ (*j* = 1,2,…, *n*), with *w*_*j*_ ∈ [0,1] and ∑_*j*=1_^*n*^ *w*_*j*_ = 1.

Let *k*(*x*) = log⁡((2 − *x*)/*x*), and then *l*(*x*) = log⁡((1 + *x*)/(1 − *x*)), *k* ^−1^(*x*) = 2/(*e* ^*x*^ + 1), and *l* ^−1^(*x*) = 1 − (2/(*e* ^*x*^ + 1)). The aggregated result using the INNWG operator in Theorem 25 can be denoted by

(31) INNWGw(A1,A2,…,An) =〈[2∏i=1ninf⁡TAiwi∏i=1n(2−inf⁡TAi)wi+∏i=1ninf⁡TAiwi,  2∏i=1nsup⁡TAiwi∏i=1n(2−sup⁡TAi)wi+∏i=1nsup⁡TAiwi],  [∏i=1n(1+inf⁡IAi)wi−∏i=1n(1−inf⁡IAi)wi∏i=1n(1+inf⁡IAi)wi+∏i=1n(1−inf⁡IAi)wi,  ∏i=1n(1+sup⁡IAi)wi−∏i=1n(1−sup⁡IAi)wi∏i=1n(1+sup⁡IAi)wi+∏i=1n(1−sup⁡IAi)wi],  [∏i=1n(1+inf⁡FAi)wi−∏i=1n(1−inf⁡FAi)wi∏i=1n(1+inf⁡FAi)wi+∏i=1n(1−inf⁡FAi)wi,  ∏i=1n(1+sup⁡FAi)wi−∏i=1n(1−sup⁡FAi)wi∏i=1n(1+sup⁡FAi)wi+∏i=1n(1−sup⁡FAi)wi]〉,

where *W* = (*w*_1_, *w*_2_,…, *w*_*n*_) is the weight vector of *A*_*j*_ (*j* = 1,2,…, *n*), with *w*_*j*_ ∈ [0,1] and ∑_*j*=1_^*n*^ *w*_*j*_ = 1.

Theorem 25 also can be proved by the mathematical induction.

Similarly, it can be proved that the INNWG operator has the same properties as the INNWA operator.

1. Idempotency: let *A*_*j*_ (*j* = 1,2,…, *n*) be a collection of INNs. If all *A*_*j*_ (*j* = 1,2,…, *n*) are equal, that is, *A*_*j*_ = *A*, for all *j* ∈ {1,2,…, *n*}, then INNWG_*w*_(*A*_1_, *A*_2_,…, *A*_*n*_) = *A*.
2. Boundedness: assume that *A*_*j*_ (*j* = 1,2,…, *n*) is a collection of INNs and *A* ^−^ = 〈min⁡_*j*_ *T*_*A*_*j*__(*x*), max⁡_*j*_ *I*_*A*_*j*__(*x*), max⁡_*j*_ *F*_*A*_*j*__(*x*)〉, *A* ^+^ = 〈max⁡_*j*_ *T*_*A*_*j*__(*x*), min⁡_*j*_ *I*_*A*_*j*__(*x*), min⁡_*j*_ *F*_*A*_*j*__(*x*)〉, for all *j* ∈ {1,2,…, *n*}, and then *A* ^−^⊆INNWG_*w*_(*A*_1_, *A*_2_,…, *A*_*n*_)⊆*A* ^+^.
3. Monotonity: assuming that *A*_*j*_ (*j* = 1,2,…, *n*) is a collection of INNs, if *A*_*j*_⊆*A*_j_*, for *j* ∈ {1,2,…, *n*}, then INNWG_*w*_(*A*_1_, *A*_2_,…, *A*_*n*_)⊆INNWG_*w*_(*A*_1_*, *A*_2_*,…, *A*_n_*).

### 4.2. Multicriteria Decision Making Method Based on the INN Aggregation Operators

Assume that there are *m* alternatives *A* = {*a*_1_, *a*_2_,…,*a*_*m*_} and *n* criteria *C* = {*c*_1_, *c*_2_,…,*c*_*n*_}, whose criterion weight vector is *W* = (*w*_1_, *w*_2_,…, *w*_*n*_), where *w*_*j*_ ≥ 0 (*j* = 1,2,…, *n*) and ∑_*j*=1_^*n*^ *w*_*j*_ = 1. Let *R* = (*a*_*ij*_)_*m*×*n*_ be the interval neutrosophic decision matrix, where *a*_*ij*_ = 〈*T*_*a*_*ij*__, *I*_*a*_*ij*__, *F*_*a*_*ij*__〉 is a criterion value, denoted by an INN, where *T*_*a*_*ij*__ indicates the truth-membership function where the alternative *a*_*i*_ satisfies the criterion *c*_*j*_, *I*_*a*_*ij*__ indicates the indeterminacy-membership function where the alternative *a*_*i*_ satisfies the criterion *c*_*j*_, and *F*_*a*_*ij*__ indicates the falsity-membership function where the alternative *a*_*i*_ satisfies the criterion *c*_*j*_.

In the following, a procedure to rank and select the most desirable alternative(s) is given.

**Step 1**

Utilize the INNWA operator or the INNWG operator to obtain the INN *y*_*i*_ for the alternatives *a*_*i*_ (*i* = 1,2,…, *m*), that is,

(32) $$\begin{array}{c} y_{i}={\text{INNWA}}_{w}\left(a_{i1},a_{i2},\ldots ,a_{in}\right) \end{array}$$

or

(33) $$\begin{array}{c} y_{i}={\text{INNWG}}_{w}\left(a_{i1},a_{i2},\ldots ,a_{in}\right). \end{array}$$

**Step 2**

Calculate the score function value *s*(*y*_*i*_), the accuracy function value *a*(*y*_*i*_), and the certainty function value *c*(*y*_*i*_) of *y*_*i*_ (*i* = 1,2,…, *m*) by Definition 18, denoted by the function matrix *F*:

(34) $$\begin{array}{c} F=\left[\begin{array}{ccc} s\left(y_{1}\right) & a\left(y_{1}\right) & c\left(y_{1}\right)\\ s\left(y_{2}\right) & a\left(y_{2}\right) & c\left(y_{2}\right)\\ \cdots & \cdots & \cdots \\ s\left(y_{m}\right) & a\left(y_{m}\right) & c\left(y_{m}\right) \end{array}\right]. \end{array}$$

**Step 3**

Construct the possibility matrix *P*_*s*_ of the score function value *s*(*y*_*i*_) as follows, according to Definition 2:

(35) $$\begin{array}{c} P_{s}=\left[\begin{array}{cccc} ps_{11} & ps_{12} & \cdots & ps_{1m}\\ ps_{21} & ps_{22} & \cdots & ps_{2m}\\ & & \vdots & \\ ps_{m1} & ps_{m2} & \cdots & ps_{mm} \end{array}\right], \end{array}$$

where *ps*_*ij*_ denotes the degree of possibility of *s*(*y*_*i*_) > *s*(*y*_*j*_), and it satisfies *ps*_*ij*_ ≥ 0, *ps*_*ij*_ + *ps*_*ji*_ = 1, and *ps*_*ii*_ = 0.5. If *ps*_*ij*_ = 0.5 (*i* ≠ *j*), then calculate the degree of possibility of *a*(*y*_*i*_) > *a*(*y*_*j*_), denoted by *pa*_*ij*_. And if *pa*_*ij*_ = 0.5 (*i* ≠ *j*), then calculate the degree of possibility of *c*(*y*_*i*_) > *c*(*y*_*j*_), denoted by *pc*_*ij*_.

**Step 4**

Get the priority of the alternatives *a*_*i*_ (*i* = 1,2,…, *m*) in accordance with *ps*_*ij*_, *pa*_*ij*_, and *pc*_*ij*_, and choose the best one, referring to Definition 20.

## 5. Illustrative Example

In this section, an example for the multicriteria decision making problem of alternatives is used as the demonstration of the application of the proposed decision making method, as well as the effectiveness of the proposed method.

Let us consider the decision making problem adapted from [33]. There is an investment company, which wants to invest a sum of money in the best option. There is a panel with four possible alternatives to invest the money: (1) *A*_1_ is a car company; (2) *A*_2_ is a food company; (3) *A*_3_ is a computer company; (4) *A*_4_ is an arms company. The investment company must make a decision according to the following three criteria: (1) *C*_1_ is the risk analysis; (2) *C*_2_ is the growth analysis; (3) *C*_3_ is the environmental impact analysis, where *C*_1_ and *C*_2_ are benefit criteria and *C*_3_ is a cost criterion. The weight vector of the criteria is given by *W* = (0.35,0.25,0.4). The four possible alternatives are to be evaluated under the above three criteria by the form of INNs, as shown in the following interval neutrosophic decision matrix *D*:

(36) D=[〈[0.4,0.5],[0.2,0.3],[0.3,0.4]〉〈[0.4,0.6],[0.1,0.3],[0.2,0.4]〉〈[0.7,0.9],[0.2,0.3],[0.4,0.5]〉〈[0.6,0.7],[0.1,0.2],[0.2,0.3]〉〈[0.6,0.7],[0.1,0.2],[0.2,0.3]〉〈[0.3,0.6],[0.3,0.5],[0.8,0.9]〉〈[0.3,0.6],[0.2,0.3],[0.3,0.4]〉〈[0.5,0.6],[0.2,0.3],[0.3,0.4]〉〈[0.4,0.5],[0.2,0.4],[0.7,0.9]〉〈[0.7,0.8],[0.0,0.1],[0.1,0.2]〉〈[0.6,0.7],[0.1,0.2],[0.1,0.3]〉〈[0.6,0.7],[0.3,0.4],[0.8,0.9]〉].

### 5.1. Procedures of Decision Making Based on INSs

**Step 1**

Utilize the INNWA operator or the INNWG operator to obtain the INNs. The aggregation results based on the INNWA operator and the INNWG operator are different, and they are calculated separately. Here, let *k*(*x*) = −log⁡*x*, which means that the operations for INNs are based on algebraic *t*-conorm and *t*-norm.

By using the INNWA operator, the alternatives matrix *A*_*WA*_ can be obtained:

(37) AWA=[〈[0.5453,0.7516],[0.1682,0.3000],[0.3041,0.4373]〉〈[0.4996,0.6634],[0.1552,0.2885],[0.3482,0.4656]〉〈[0.3950,0.5627],[0.2000,0.3366],[0.4210,0.5533]〉〈[0.6383,0.7397],[0.0000,0.2071],[0.2297,0.4040]〉].

With the INNWG operator, the alternatives matrix *A*_*WG*_ is shown as follows:

(38) AWG=[〈[0.5004,0.6620],[0.1761,0.3000],[0.3195,0.4422]〉〈[0.4547,0.6581],[0.1861,0.3371],[0.5405,0.6786]〉〈[0.3824,0.5578],[0.2000,0.3419],[0.5012,0.7070]〉〈[0.6333,0.7335],[0.1555,0.2570],[0.5069,0.6632]〉].

**Step 2**

Calculate the score function value, accuracy function value, and certainty function value.

To the alternatives matrix *A*_*WA*_, by using Definition 18, the function matrix of *A*_*WA*_ can be obtained:

(39) FWA=[[1.8080,2.2793][0.2412,0.3143][0.5453,0.7516][1.7455,2.1600][0.1514,0.1978][0.4996,0.6634][1.5051,1.9417][−0.0260,0.0094][0.3950,0.5627][2.0272,2.5100][0.3357,0.4086][0.6383,0.7397]].

To the alternatives matrix *A*_*WG*_, by using Definition 20, the function matrix of *A*_*WG*_ is shown as follows:

(40) FWG=[[1.7582,2.1664][0.1809,0.2198][0.5004,0.6620][1.4390,1.9315][−0.0858,−0.0205][0.4547,0.6581][1.3335,1.8566][−0.1492,−0.1188][0.3824,0.5578][1.7131,2.0711][0.0703,0.1264][0.6333,0.7335]].

**Step 3**

Construct the possibility matrix. Each interval number is compared to all interval numbers. Referring to Definition 2, the possibility matrix of the score function value *s*(*y*_*i*_) can be obtained. For *F*_*WA*_,

(41) $$\begin{array}{c} P_{s\_WA}=\left[\begin{array}{cccc} 0.5 & 0.6498 & 0.8527 & 0.2642\\ 0.3974 & 0.5 & 0.7695 & 0.1480\\ 0.1473 & 0.2305 & 0.5 & 0\\ 0.7358 & 0.8520 & 1 & 0.5 \end{array}\right]. \end{array}$$

And, for *F*_*WG*_,

(42) $$\begin{array}{c} p_{s\_WG}=\left[\begin{array}{cccc} 0.5 & 0.8076 & 0.8943 & 0.5916\\ 0.1924 & 0.5 & 0.5888 & 0.2568\\ 0.1057 & 0.4112 & 0.5 & 0.1629\\ 0.4084 & 0.7432 & 0.8371 & 0.5 \end{array}\right]. \end{array}$$

It is obvious that *ps*_*ij*_ ≠ 0.5 (*i* ≠ *j*), so there is no need to compute *pa*_*ij*_ and *pc*_*ij*_.

**Step 4**

Get the priority of the alternatives and choose the best one.

According to Definition 20 and results in Step 3, for *A*_*WA*_, we have *A*_1_≻*A*_2_, *A*_1_≻*A*_3_, *A*_2_≻*A*_3_, *A*_4_≻*A*_1_, and *A*_4_≻*A*_2_. Therefore, the ranking of the four alternatives is *A*_4_, *A*_1_, *A*_2_, and *A*_3_. Obviously, *A*_4_ is the best alternative.

Similarly, for *A*_*WG*_, we have *A*_1_≻*A*_2_, *A*_1_≻*A*_3_, *A*_1_≻*A*_4_, *A*_2_≻*A*_3_, *A*_4_≻*A*_2_, and *A*_4_≻*A*_3_. Therefore, the ranking of the four alternatives is *A*_1_, *A*_4_, *A*_2_, and *A*_3_. Obviously, *A*_1_ is the best alternative.

When *k*(*x*) = log⁡((2 − *x*)/*x*), for *A*_*WA*_, the ranking of the four alternatives is still *A*_4_, *A*_1_, *A*_2_, and *A*_3_, as well as the ranking *A*_1_, *A*_4_, *A*_2_, and *A*_3_ for *A*_*WG*_.

### 5.2. Comparison Analysis and Discussion

In order to validate the feasibility of the proposed decision making method based on the INN aggregation operators, a comparison analysis will be conducted. In Section 5.1, the same example adapted from [33] for the multicriteria decision making problem is demonstrated based on the INN aggregation operators. This analysis will be based on the same illustrative example.

There is no consensus on the best way to sequence INNs. Ye proposed the similarity measures between INSs based on the relationship between similarity measures and distances and utilized the similarity measures between each alternative and the ideal alternative to establish a multicriteria decision making method for INSs in [33]. By contrast, we present the aggregation operators for INNs and put forward a method for multicriteria decision making by means of the aggregation operators.

With the same example, [33] gave two rankings of the four alternatives with different similarity measures. The first one is *A*_4_, *A*_2_, *A*_3_, and *A*_1_. The second one is *A*_2_, *A*_4_, *A*_3_, and *A*_1_. Unlike the results in [33], we obtained the ranking sequences as *A*_4_, *A*_1_, *A*_2_, *A*_3_ and *A*_1_, *A*_4_, *A*_2_, and *A*_3_. Obviously, the results in [33] conflict with ours in this paper. And the difference mainly lies in the position of *A*_1_.

Here, for convenience, the decision matrix *D* in Section 5 is denoted by

(43) $$\begin{array}{c} D=\left[\begin{array}{ccc} a_{11} & a_{12} & a_{13}\\ a_{21} & a_{22} & a_{23}\\ a_{31} & a_{32} & a_{33}\\ a_{41} & a_{42} & a_{43} \end{array}\right]. \end{array}$$

Certainly, the alternatives *A*_1_ can be obtained by the decision vector $\left(\begin{array}{ccc} a_{11} & a_{12} & a_{13} \end{array}\right)$ with the associated weight vector *W* = (0.35,0.25,0.4). Firstly, consider *A*_1_ and *A*_3_. As can be seen from the decision matrix *D*, the truth-membership, the indeterminacy-membership, and the falsity-membership satisfies

(44) $$\begin{array}{c} I_{a_{11}}=I_{a_{31}},\;\;F_{a_{11}}=F_{a_{31}}. \end{array}$$

And, with Definition 2, *p*(*T*_*a*_11__ > *T*_*a*_31__) = 0.5, so *T*_*a*_11__ = *T*_*a*_31__. Therefore, *a*_11_ = *a*_31_. Similarly, it can obtained that *a*_13_ = *a*_33_ significantly. And by Definitions 18 and 20, *a*_12_ < *a*_32_ with a bit difference; that is, *a*_12_ is close to *a*_32_, so that, with the weighted vector *W* = (0.35,0.25,0.4), *A*_1_≻*A*_3_. Thus there is a conflict of sequences of *A*_1_ and *A*_3_ in [33].

Similarly, it is obvious that *a*_21_ < *a*_41_, *a*_22_ < *a*_42_, and *a*_23_ < *a*_43_, so that with the associated weight vector *W* = (0.35,0.25,0.4), *A*_1_≺*A*_4_, which is not coordinated with the ranking of *A*_2_, *A*_4_, *A*_3_, and *A*_1_ in [33], while the sequences of *A*_1_, *A*_2_ and *A*_1_, *A*_3_ obtained by the method in this paper are consistent with the realities. Here are the reasons for this. The difference between INSs is distorted. In the similarity measures in [33], the distances between INSs are calculated firstly and the difference was amplified in the results because of criteria weights. This causes the distortion of similarity between an alternative and the ideal alternative. In addition, the ranking of all alternatives was determined by the similarity, so that the degree of distortion can not be reduced. However, the difference between INSs in the method proposed in this paper was reserved to the final calculation. Combining the factors above, the final result produced by the method proposed in this paper is more precise and reliable than the result produced in [33].

## 6. Conclusion

INSs can be applied in addressing problems with uncertain, imprecise, incomplete, and inconsistent information existing in real scientific and engineering applications. However, as a new branch of NSs, there is no enough research about INSs. In particular, the existing literatures do not put forward the aggregation operators and multicriteria decision making method for INSs. Based on the related research achievements in IVIFSs, we defined the operations of INSs. And the approach to compare INNs was proposed. In addition, the aggregation operators of INNWA and INNWG were given. Thus, a multicriteria decision making method is established based on the proposed operators. Utilizing the comparison approach, the ranking of all alternatives can be determined and the best one can be easily identified as well. The illustrative example demonstrates the application of the proposed decision making method. Although there is no consensus on the best way to sequence INNs, compared to the multicriteria decision making method for INSs in [33], the illustrative example shows that the final result produced by the method proposed in this paper is more precise and reliable than the result produced in [33]. In this way, the method proposed in this paper can provide a reliable basis for INSs.
